# Supplementary material for: A study on the impact of tourism destination image and local attachment on the revisit intention: The moderating effect of perceived risk
Source: PLoS One. 2024 Jan 5;19(1):e0296524. doi: 10.1371/journal.pone.0296524 (PMC10769066; doi:10.1371/journal.pone.0296524)
Supplement: S1 Appendix — (DOCX) [file pone.0296524.s001.docx]

**Appendix**

**Questionnaire**

Dear ladies/gentlemen，

Thank you very much for participating in the survey amidst your busy schedule! Please truthfully fill in the measurement of each variable and fill in personal information based on your needs and willingness after the release of epidemic prevention and control, and mark “√“” on the options. This questionnaire is filled out anonymously, and the survey results will only be used for scientific research and not for any commercial purposes. Please rest assured and objective in filling it out. Thank you very much for your participation!

**Part 1: Personal information**

1. Your gender is: ① Male ② Female

2. You come from: ① First-tier cities ② Second-tier cities ③ Third-tier cities and other cities ④ County towns and townships ⑤ Rural areas

3. Your age is: ① Under 22 years old ② 23-35 years old ③ 36-59 years old ④ Over 60 years old

4. Your educational background: ① Secondary school and below ② College degree ③ Bachelor ④Master's and PhD

5. Your single visit time: ① Under 6 hours ② 6 -12 hours ③ More than 12 hours

6. Your number of visit: ① 1, ② 2, ③ 3, ④ 4 or more

**Part 2: Measurement items for each variable**

**Tourism destination image**

1. Guilin Lijiang Scenic Area is very beautiful( )

①Very consistent ② Consistent ③ Basically consistent ④Not very consistent ⑤ Not consistent

1. Guilin Lijiang Scenic Area makes me relax ( )

①Very consistent ② Consistent ③ Basically consistent ④Not very consistent ⑤ Not consistent

3. The equipment in Guilin Lijiang Scenic Area is very complete ( )

①Very consistent ② Consistent ③ Basically consistent ④Not very consistent ⑤ Not consistent

4.The service quality of Guilin Lijiang Scenic Area is high ( )

①Very consistent ② Consistent ③ Basically consistent ④Not very consistent ⑤ Not consistent

5. Guilin Lijiang Scenic Area is safe and reliable（ ）

①Very consistent ② Consistent ③ Basically consistent ④Not very consistent ⑤ Not consistent

6.Guilin Lijiang Scenic Area is pleasant（ ）

①Very consistent ② Consistent ③ Basically consistent ④Not very consistent ⑤ Not consistent

**Nostalgia emotion**

7. The tourism experience in Guilin Lijiang Scenic Area has left me unforgettable（ ）

①Very consistent ② Consistent ③ Basically consistent ④Not very consistent ⑤ Not consistent

8.The tourism experience in Guilin Lijiang Scenic Area makes me feel happy（ ）

①Very consistent ② Consistent ③ Basically consistent ④Not very consistent ⑤ Not consistent

9.The tourism experience in Guilin Lijiang Scenic Area reminds me of my loved ones（ ）

①Very consistent ② Consistent ③ Basically consistent ④Not very consistent ⑤ Not consistent

10.The tourism experience in Guilin Lijiang Scenic Area has made me reminisce about my past life（ ）

①Very consistent ② Consistent ③ Basically consistent ④Not very consistent ⑤ Not consistent

11.The tourism experience in Guilin Lijiang Scenic Area has given me the motivation to live（ ）

①Very consistent ② Consistent ③ Basically consistent ④Not very consistent ⑤ Not consistent

**Local attachment**

12.Guilin Lijiang Scenic Area has given me a sense of belonging（ ）

①Very consistent ② Consistent ③ Basically consistent ④Not very consistent ⑤ Not consistent

13.My heart still stays in Guilin Lijiang Scenic Area（ ）

①Very consistent ② Consistent ③ Basically consistent ④Not very consistent ⑤ Not consistent

14. I like Guilin Lijiang Scenic Area（ ）

①Very consistent ② Consistent ③ Basically consistent ④Not very consistent ⑤ Not consistent

15.I acknowledge the culture of Guilin Lijiang Scenic Area（ ）

①Very consistent ② Consistent ③ Basically consistent ④Not very consistent ⑤ Not consistent

16.I acknowledge the services of Guilin Lijiang Scenic Area（ ）

①Very consistent ② Consistent ③ Basically consistent ④Not very consistent ⑤ Not consistent

**Perceived risk**

17. I’m worried that I don’t have time to travel（ ）

①Very consistent ② Consistent ③ Basically consistent ④Not very consistent ⑤ Not consistent

18. The cost of Guilin Lijiang Scenic Area is too high（ ）

①Very consistent ② Consistent ③ Basically consistent ④Not very consistent ⑤ Not consistent

19.The service quality of Guilin Lijiang Scenic Area is not high（ ）

①Very consistent ② Consistent ③ Basically consistent ④Not very consistent ⑤ Not consistent

20.Incomplete safety measures in Guilin Lijiang Scenic Area（ ）

①Very consistent ② Consistent ③ Basically consistent ④Not very consistent ⑤ Not consistent

21.The food in Guilin Lijiang Scenic Area is not what I like（ ）

①Very consistent ② Consistent ③ Basically consistent ④Not very consistent ⑤ Not consistent

**Revisit intention**

22. I am willing to revisit the Lijiang Scenic Area in Guilin（ ）

①Very consistent ② Consistent ③ Basically consistent ④Not very consistent ⑤ Not consistent

23. I am willing to revisit the newly developed scenic spots of Guilin Lijiang Scenic Area（ ）

①Very consistent ② Consistent ③ Basically consistent ④Not very consistent ⑤ Not consistent

24.I will recommend friends to visit the Lijiang Scenic Area in Guilin（ ）

①Very consistent ② Consistent ③ Basically consistent ④Not very consistent ⑤ Not consistent
