# Supplementary material for: A study on the impact of tourism destination image and local attachment on the revisit intention: The moderating effect of perceived risk
Source: PLoS One. 2024 Jan 5;19(1):e0296524. doi: 10.1371/journal.pone.0296524 (PMC10769066; doi:10.1371/journal.pone.0296524)
Supplement: Attachment — Submitted filename: 2023-10-21-reviewer-final.docx [file pone.0296524.s003.docx]

In review of this manuscript, several concerns have been identified, detailed as follows:

1. The abstract does not adequately engage with existing literature to highlight the novelty of the manuscript.

2. The literature review section appears to be a mere enumeration of existing works, failing to underscore the innovative aspects of the manuscript.

3. The research design section lacks rigor. The questionnaire data presented do not sufficiently establish causality to yield empirical findings.

4. The data analysis section would benefit from a more comprehensive discussion.

5. The conclusion section appears to overstate its novelty, particularly in terms of the research scenarios. Additionally, the manuscript would benefit from refinements in language and formatting.
